# Breakthrough in Blastic Plasmacytoid Dendritic Cell Neoplasm Cancer Therapy Owing to Precision Targeting of CD123

**DOI:** 10.3390/ijms25031454

**Published:** 2024-01-25

**Authors:** Serena Zanotta, Domenico Galati, Rosaria De Filippi, Antonio Pinto

**Affiliations:** 1 Hematology-Oncology and Stem-Cell Transplantation Unit, Department of Onco-Hematology and Innovative Diagnostics, Istituto Nazionale Tumori-IRCCS-Fondazione G. Pascale, 80131 Napoli, Italy; s.zanotta@istitutotumori.na.it (S.Z.); a.pinto@istitutotumori.na.it (A.P.); 2 Department of Clinical Medicine and Surgery, Università degli Studi di Napoli Federico II, 80131 Napoli, Italy; rdefillp@unina.it

**Keywords:** BPDCN, dendritic cells, pDC, cancer therapy, immunotherapy

## Abstract

Blastic plasmacytoid dendritic cell neoplasm (BPDCN) is a rare and aggressive hematologic cancer originating from the malignant transformation of plasmacytoid dendritic cell precursors. This malignancy progresses rapidly, with frequent relapses and a poor overall survival rate, underscoring the urgent need for effective treatments. However, diagnosing and treating BPDCN have historically been challenging due to its rarity and the lack of standardized approaches. The recognition of BPDCN as a distinct disease entity is recent, and standardized treatment protocols are yet to be established. Traditionally, conventional chemotherapy and stem cell transplantation have been the primary methods for treating BPDCN patients. Advances in immunophenotyping and molecular profiling have identified potential therapeutic targets, leading to a shift toward CD123-targeted immunotherapies in both clinical and research settings. Ongoing developments with SL-401, IMGN632, CD123 chimeric antigen receptor (CAR) T-cells, and bispecific antibodies (BsAb) show promising advancements. However, the therapeutic effectiveness of CD123-targeting treatments needs improvement through innovative approaches and combinations of treatments with other anti-leukemic drugs. The exploration of combinations such as CD123-targeted immunotherapies with azacitidine and venetoclax is suggested to enhance antineoplastic responses and improve survival rates in BPDCN patients. In conclusion, this multifaceted approach offers hope for more effective and tailored therapeutic interventions against this challenging hematologic malignancy.

## 1. Introduction

The neoplastic proliferation of plasmacytoid dendritic cells (pDCs) is known to give rise to BPDCN, as well as to the clonal expansion of mature pDCs [1]. First identified as a unique category in 1994, BPDCN is a rare and aggressive hematologic cancer originating from the malignant transformation of the non-activated precursors of pDC. This malignancy represents less than 1% of all hematologic tumor cases [2,3]. Its primary impact is observed in older adults, commonly surfacing in the sixth decade of life. Significantly, there is a higher prevalence among males, and individuals of Caucasian descent are slightly more affected than those of other demographics. Beyond its epidemiological profile, BPDCN exhibits a set of unique clinical, morphological, and immunophenotypic features, setting it apart from other hematopoietic neoplasms [4]. The nomenclature evolved in 2016 when the World Health Organization (WHO) classified BPDCN as a distinct malignancy within myeloid neoplasms and acute leukemia [5,6]. While the specific developmental pathway of pDC malignancies remains a subject of debate, prevailing evidence supports that the malignant proliferation of pDC precursors is specifically involved in the pathogenesis of BPDCN [3,7,8]. To comprehend this, it is essential to delve into the classification of DCs and their functional subtypes.

### 1.1. Dendritic Cell Classification

Dendritic cells (DCs), originating from bone marrow, are a diverse group found in the circulation, lymphoid organs, and various tissues. Modern classification recognizes two principal functional subtypes: conventional DCs (cDCs) and pDCs [9,10]. In the bloodstream, there are cDCs that express CD11c, including cDC2 CD1c^+^ and cDC1 CD141^+^ cells. Plasmacytoid dendritic cells, characterized by CD123^+^ and CD303^+^ markers, are also present but do not express CD11c. Although pDCs make up a minor fraction of the DC population, they predominantly reside in the blood and lymphoid tissues [9,10,11]. These cells are renowned for their robust antiviral defense capabilities, primarily through the secretion of type I interferon (IFN). Despite their antiviral function, pDCs also play a role in immune tolerance, as evidenced by their ability to stimulate regulatory T cells (Tregs) and T cells producing interleukin (IL)-10, indicating a potentially dualistic role in immune response regulation [12,13,14].

### 1.2. Clinical Manifestations

The importance of accurately identifying and understanding BPDCN is emphasized to enhance clinical management and treatment strategies for this specific subset of hematologic malignancies. BPDCN patients typically initially show skin involvement, which can extend to affect bone marrow (BM), peripheral blood (PB), and lymph nodes (LN). Extra-cutaneous manifestations often appear at diagnosis, particularly in regional LNs, progressing to involve PB and BM as the disease advances [2,15]. This malignancy is characterized by rapid progression, frequent relapses, and poor overall survival (OS), highlighting the need for effective treatment strategies [16].

Historically, diagnosing and treating BPDCN have been challenging due to its rarity and the lack of standardized approaches. It predominantly affects the elderly, with a noticeable male prevalence, and most patients experience cytopenias, lymphadenopathy, and/or splenomegaly [2]. As leukemic presentation is infrequent, occurring in less than 1% of acute leukemia cases, only 10% of those initially presenting with isolated skin lesions progress rapidly to leukemia [2,17,18]. Interestingly, a minority of cases present with leukemia but without skin involvement [15]. Advances in immunophenotyping and molecular profiling have uncovered potential therapeutic targets.

### 1.3. Diagnosis of BPDCN

The accurate identification and classification of BPDCN require a combination of clinical expertise and precise diagnostic laboratory tests due to its clinical and biological heterogeneity. Immunophenotypic criteria, using either flow cytometry (FC) or immunohistochemistry (IHC), are the primary methods for identifying this neoplasm [19].

Flow cytometry is considered a crucial diagnostic tool for BPDCN due to its objectivity and quantitative precision. It has the ability to detect small populations of cells with abnormal phenotypes among normal leukocytes, which is particularly important for accurately diagnosing BPDCN. FC outperforms IHC in diagnosing BPDCN because it can simultaneously detect multiple antigens, including those that are not routinely examined via IHC. This heightened sensitivity is essential in overcoming the challenges associated with accurately diagnosing BPDCN. However, diagnosing BPDCN is challenging due to its immunophenotypic heterogeneity, which often leads to features overlapping other hematologic neoplasms such as NK/T cell leukemia/lymphoma, cutaneous T cell lymphoma, and acute myelomonocytic leukemia expressing CD4 or CD56 [20]. Moreover, the initial assumption that BPDCN derived from NK cells based on the absence of common lineage markers and the expression of CD4 and CD56 has been reconsidered. Further studies have revealed the intricate nature of BPDCN and its shared characteristics with pDCs. These include the absence of T cell receptor and immunoglobulin heavy chain gene rearrangements, as well as the production of IFN-alfa (IFN-α) and Th2 polarization following Interleukin-3 (IL-3) induction [21,22,23,24].

In conclusion, successfully addressing the diagnostic challenges posed by BPDCN requires a deep understanding of its immunophenotypic complexities, the strategic use of advanced diagnostic methods like flow cytometry, and staying informed about evolving insights into its cellular origins and molecular characteristics.

### 1.4. Immunophenotype of BPDCN

The diagnosis of BPDCN hinges on discerning specific immunophenotypic features within neoplastic cells [25]. Typically, BPDCN cells coexpress CD4 and CD56, in addition to CD34, CD36, and pDC-associated antigens like CD123 and CD303. Rare instances may involve an aberrant expression of CD5, CD7, CD33, TdT, and CD79a. The presence of detrimental lineage-specific antigens (Lin-) has also been observed [20,24,26].

A recent study has categorized BPDCN into three maturation-associated subgroups based on the expression of CD34 and CD117 in pDCs. In stage 1, CD34 is expressed in some immature blasts; in stage 2, blast cells partially express CD117, while CD34 is negative; in stage 3, both CD34 and CD117 are negative in blast cells. These distinctive maturation profiles contribute to the diverse clinical presentations and laboratory characteristics of BPDCN.

While the origin and development of pDC malignancies remain debated, contemporary evidence leans towards considering BPDCN cells the malignant counterparts of precursor pDCs [3,7].

### 1.5. Molecular and Genetic Analyses

Molecular and genetic analyses play a crucial role in the detailed characterization of BPDCN by offering valuable diagnostic and prognostic insights. Cytogenetic techniques, such as karyotyping and fluorescence in situ hybridization (FISH) have been instrumental in identifying frequent chromosomal anomalies associated with BPDCN. Notable abnormalities include deletions or rearrangements of chromosomes 5q, 12p, and 13q, and mutations in the TP53 gene. Further refinement in detection methods comes from next-generation sequencing (NGS), which sheds light on specific genetic mutations. Alterations in genes like Tet methyl cytosine dioxygenase (TET2), ASXL transcriptional regulator 1 (ASXL1), and DNA methyltransferase 3 alpha (DNMT3A) are commonly reported in BPDCN [24,25,26,27,28]. Moreover, chromosomal changes commonly linked to BPDCN include anomalies in regions 5q, 6q, monosomy 9, 12p, 13q, and 15q [18,26,27,28]. In particular, some of these chromosomal deletions prominently affect key genes involved in cell cycle regulation (CDKN2A/CDKN2B and CDKN1B), tumor suppression (RB1, TP53, and PTEN), transcription factors (IKZF1 and ETV6), and other relevant genes (NR3C1 and LATS2) [28].

## 2. Treatment of BPDCN

### 2.1. Treatment Strategies for BPDCN

The recognition of BPDCN as a distinct disease entity is a recent development, and due to its rarity, the establishment of standardized treatment protocols is yet to be achieved. Traditionally, conventional chemotherapy has been the primary method of treating BPDCN.

### 2.2. Conventional Chemotherapy

Chemotherapy regimens originally designed for treating acute leukemia have shown varying degrees of success in inducing sustained clinical responses in BPDCN patients. Regimens that combine anthracycline-based induction therapies with high-dose cytarabine have produced encouraging remission rates. In clinical settings, programs such as HyperCVAD (cyclophosphamide, vincristine, doxorubicin, and dexamethasone) and CHOP (cyclophosphamide, doxorubicin, vincristine, and prednisone) have been employed [2,29]. The determination of the most effective treatment plan, including the regimen and length of therapy, remains contentious, largely due to the scarcity of comprehensive clinical trials and variability in patient profiles.

### 2.3. Stem Cell Transplantation

In selected BPDCN patients, allogeneic stem cell transplantation (allo-SCT) offers a potentially curative approach and is considered the gold-standard consolidation therapy in patients who attain remission and are fit for the procedure. This, however, often excludes the majority of patients over the age of 65 or those with compromised clinical conditions and comorbidities [5,30,31]. Evidence indicates that conducting allo-SCT during the first complete remission (CR1) or after securing a durable remission through induction chemotherapy can enhance OS rates and decrease relapse risks [4,29]. Nevertheless, refining patient selection criteria, deciding on the optimal timing for transplantation, and determining effective conditioning regimens are all active fields of investigation.

## 3. CD123 Molecule

**Figure 1:**
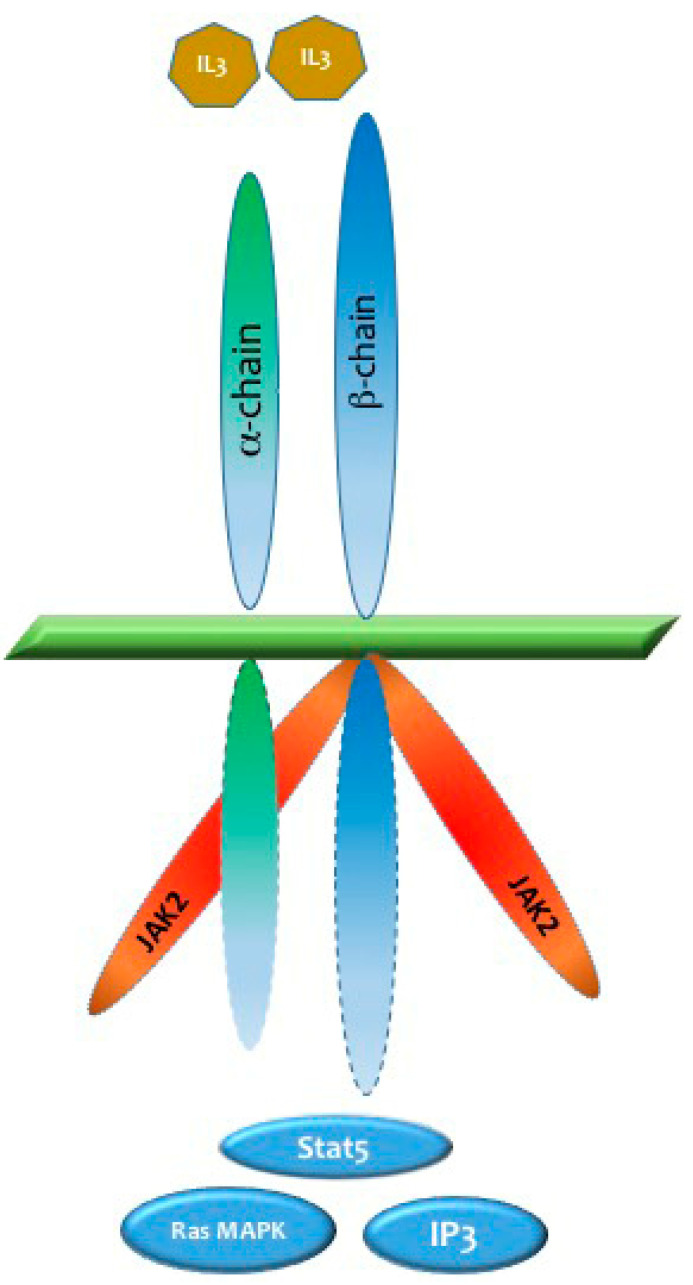
Molecular mechanisms of IL-3 receptor (CD123) activation. CD123, as the alpha subunit of the IL-3 receptor, guides hematopoietic cell maturation. When bound to IL-3, it activates the beta chain, creating a dynamic IL-3 receptor heterodimer. This receptor orchestrates crucial signaling pathways for anti-apoptotic and cell-proliferative signals.

CD123 has emerged as a promising therapeutic target for patients with BPDCN [32]. This cell surface molecule is expressed on pDCs, basophils, monocytes, and eosinophils, with a noted overexpression in a variety of hematological malignancies [33]. A pivotal discovery in the understanding of BPDCN was the identification of CD123 overexpression in nearly all cases of the disease [34,35,36]. High levels of CD123 expression were detected in up to 100% of the samples analyzed through flow cytometry, not just in BPDCN but also in acute myeloid leukemia (AML), B-cell acute lymphoblastic leukemia (B-ALL), hairy cell leukemia (HCL), myelodysplastic syndromes (MDS), and chronic eosinophilic leukemia [37,38,39,40]. As the alpha subunit of the interleukin-3 receptor (IL-3Ra), CD123 plays a fundamental role in the maturation of hematopoietic cells [41]. The binding of IL-3 to IL-3Ra activates the beta chain, forming a heterodimeric IL-3 receptor that transmits vital signals, leading to the activation of multiple pathways, including Janus kinase/signal transducers and activators of transcription (JAK/STAT), Ras-mitogen-activated protein kinase (Ras/MAPK), and phosphoinositide 3-kinase (PI3K) (Figure 1). These pathways are particularly important for anti-apoptotic and cell-proliferative signaling [41].

**Figure 2:**
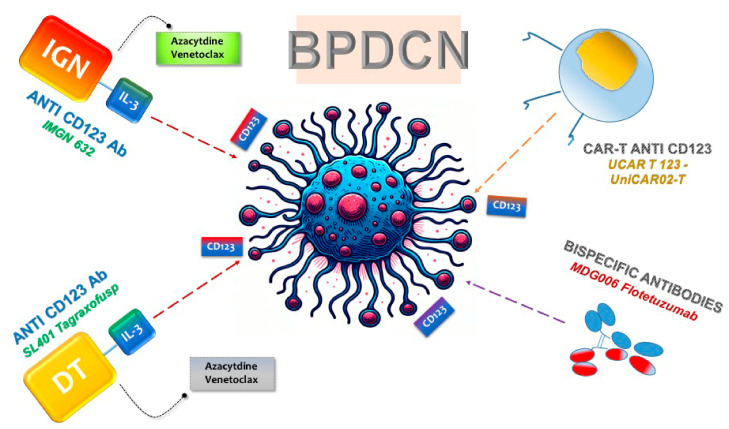
Targeting of CD123 in BPDCN Therapies. The figure illustrates the significant up regulation of CD123 on neoplastic cells, leading to the development of targeted therapeutic strategies. Monoclonal antibodies, BsAbs, and cellular therapies, including chimeric antigen receptor (CAR) T cells, have advanced to early-phase clinical trials for BPDCN. These therapies leverage the specificity for CD123 to selectively target and treat tumor cells.

The pronounced overexpression of CD123 on neoplastic cells has paved the way for the design of targeted therapeutic agents aimed at this molecule, such as monoclonal antibodies, bispecific T cell antibodies (BsAbs), and cellular therapies like chimeric antigen receptor (CAR) T cells (Figure 2). These therapies have progressed to early-phase clinical trials for BPDCN, utilizing the specificity for CD123 to selectively target tumor cells [42,43,44].

### 3.1. ANTI-CD123 Tagraxofusp

Preliminary pilot studies on a CD123-targeted agent have unveiled a pioneering recombinant protein medication. This cutting-edge therapeutic, known as Diphtheria toxin (DT)-IL3, incorporates a modified DT combined with recombinant human IL-3 [45] (Figure 2). Initially employed in patients with MDS and AML, including a subset with BPDCN [46], DT-IL3 underwent further examination in a pilot study by Frankel et al. Specifically focusing on BPDCN patients, the targeted therapy was subsequently renamed SL-401 (Tagraxofusp, Stemline Therapeutics, New York, NY, USA) [43].

In a phase I–II clinical trial involving 11 patients with relapsed/refractory BPDCN, Tagraxofusp demonstrated significant efficacy. Treatment-naïve patients exhibited major responses in 90% of cases, with 72% of these responses culminating in CR. Commonly reported adverse events included thrombocytopenia, hypoalbuminemia, and elevated liver function tests [47].

This promising outcome led to a prospective multi-institutional phase II clinical study, encompassing 47 subjects with untreated or relapsed BPDCN. Among the 29 previously untreated patients, a remarkable 90% overall response rate was observed, with 45% of them qualifying for hematopoietic stem cell transplantation (HSCT). The 2-year OS rate reached 52%. Among the fifteen patients who had received prior treatment, the response rate reached 67%, with a median OS time of 8.5 months [48].

Significant advancements in terms of Tagraxofusp in BPDCN treatment were evident in both in vitro and in vivo models, displaying potent antitumor activity at minimal concentrations [48,49]. Consequently, it received approval from the United States Federal Drug Administration for patients aged 2 and older with previously untreated or relapsed/refractory BPDCN, followed by approval in the European Union for adults as a first-line treatment in January 2021 [48,49].

While Tagraxofusp has proven efficacious in reducing disease burden, it does come with a notable side effect profile, most frequently encountered in the first cycle of therapy. Notable side effects include hepatotoxicity (88%), thrombocytopenia (49%), and capillary leak syndrome (CLS) (19%) [48,50]. Despite facing numerous obstacles, the effectiveness of CD123-targeted therapy in eliciting a clinical response establishes its role as a crucial element in the management of BPDCN for patients who are undergoing initial treatment as well as those with refractory disease.

### 3.2. Resistance to Tagraxofusp—Combination Therapy

Resistance in cancer cells does not stem from CD123 loss but rather from their ability to resist the diphtheria toxin, facilitated by the DNA methylation-induced silencing of genes in the diphthamide synthesis pathway [51,52,53,54]. Azacytidine has proven effective in reversing diphthamide silencing, thus addressing Tag resistance [55] (Figure 2). Additionally, post-Tag treatment, surviving cancer cells exhibit heightened expression of the anti-apoptotic protein BCL2. Combating these adaptations and boosting sensitivity involves the strategic use of the BCL2 inhibitor Venetoclax alongside conventional chemotherapy [56] (Figure 2).

To address these complexities, ongoing clinical trials (NCT03113643) are exploring Tag in combination with Azacitidine and Venetoclax for patients with BPDCN, AML, and MDS. Emerging data suggest a synergistic effect, leading to enhanced cell death. Azacytidine’s impact on DNA methylation is crucial, reactivating Tag sensitivity in cells that manage to escape, often by downregulating the diphthamide pathway [56].

Ongoing and completed clinical trials are illustrated in Table 1.

### 3.3. IMGN632

IMGN632 stands as a noteworthy advancement in cancer therapeutics, being a humanized IgG1 monoclonal antibody meticulously engineered for CD123, coupled with a pioneering DNA-alkylating indolinobenzodiazepine pseudodimer (IGN) (Figure 2). This antibody–drug conjugate selectively binds to CD123-expressing cells, undergoing internalization and releasing FGN849, a potent DNA-alkylating agent capable of inducing cell lysis and apoptosis (Figure 2). Notably, IMGN632 exhibits robust efficacy against the BPDCN cell line CAL1 and demonstrates significant activity in vivo within patient-derived xenograft (PDX) models of BPDCN [57,58]. Further supporting its targeted action, studies reveal that decreased CD123 expression in normal hematopoietic stem and progenitor cells correlates with reduced sensitivity to IMGN632 [57,58].

In a recent phase I/II clinical study (NCT03386513), IMGN632 was evaluated as a single agent in patients with relapsed/refractory AML, BPDCN, or other CD123 hematologic malignancies. Among the 23 patients with relapsed/refractory BPDCN, administered at a dose level of 0.045 mg/kg via intravenous infusion once every three weeks, 30% demonstrated an objective response with a composite CR rate of 22% (two CR, two clinical CR (CRc), one CRi, and two partial response). Impressively, the duration of response for the CR/CRc patients ranged between 3 and 9 months, even without undergoing stem cell transplantation. IMGN632 showcased a favorable safety profile, with no grade 3 or higher adverse events reported in more than one patient. The most common adverse events included nausea, peripheral edema, and infusion-related reactions. Notably, in contrast to Tagraxofusp, no instances of CLS were observed [59].

In light of this promising preliminary evidence, IMGN632 received a breakthrough therapy designation (BTD) from the US Food and Drug Administration in 2020, specifically for the treatment of BPDCN. This designation bestows priority review status upon the agent and its manufacturer for future evaluation by the regulatory agency, marking a significant step forward in the pursuit of effective therapies for BPDCN.

### 3.4. Combination Therapy

IMGN632 is undergoing further investigation in a phase Ib/II study for patients with AML, where it is being assessed in combination with azacitidine, venetoclax, or a combination of both (NCT04086264). The outcomes of this study, encompassing both safety and efficacy data, may potentially pave the way for exploring IMGN632 combinations in the context of BPDCN in the future.

Ongoing and completed clinical trials are illustrated in Table 1.

## 4. Anti-CD123 Chimeric Antigen Receptors T Cell in BPDCN

Autologous T cells engineered to express CAR represent a promising avenue for the treatment of diverse tumors, eliciting a robust T cell immune response that specifically targets and eliminates cancerous cells [60,61,62]. Notably, CAR T cell immunotherapy targeting the pan-B-cell antigen CD19 has demonstrated significant success, particularly in achieving high remission rates among patients with ALL and non-Hodgkin lymphoma (NHL), leading to accelerated FDA approvals in 2017 [63,64]. In the quest for effective T cell-based immunotherapy, CD123 emerges as an attractive target. Preclinical studies have shown that anti-CD123 CAR-T therapy holds substantial promise in the treatment of BPDCN [65]. To mitigate potential adverse effects, such as cytokine release syndrome (CRS) resulting from a robust immune response, these trials have incorporated safety switches in the form of various CAR designs [66,67].

Specifically, T cells from BPDCN patients transduced with CD28/4-1BB CD123 CAR have demonstrated efficacy in vitro, successfully eliminating autologous BPDCN blasts and reducing the BPDCN blast burden in vivo, without causing significant on-target/off-tumor toxicity effects [68]. A recent development in the field is the emergence of TCRαβ-negative allogeneic CAR T cells, often referred to as “universal” CAR T cells (Figure 2). Notably, UCAR T 123, composed of allogeneic T cells from healthy donors expressing anti-CD123 CAR and edited using transcription activator-like effector nuclease (TALEN), presents a promising candidate for treating relapsed/refractory AML and BPDCN (Figure 2) [67].

UCART123 cell therapy, as a salvaging method for BPDCN patients unable to harvest normal T cells for CAR T generation, has demonstrated success in xenograft mouse models with primary patient-derived BPDCN. However, challenges may arise due to the potential loss of the CD123 antigen, as detected in some BPDCN cases. In summary, these results provide a preclinical proof-of-principle that allogeneic UCART123 cells have potent anti-BPDCN activity [67].

Clinical trials, such as NCT03203369 and NCT04109482, are actively assessing the efficacy of anti-CD123 CAR T cells for BPDCN treatment, although the submission of results is still pending, according to ClinicalTrials.gov.

In addition to UCAR T 123, several other CAR-T products are currently under investigation in clinical trials. The noteworthy ones among them are MB-102 (NCT04109482 and NCT02159495) and UniCAR02-T (NCT04230265), both targeted for relapsed or refractory CD123 hematologic neoplasms and BPDCN patients [65,69,70] (Figure 2). Results from two-phase clinical studies (NCT04109482 and NCT02159495) using MB-102 have shown initial responses in four out of seven patients, including one with BPDCN. Meanwhile, a phase I study (NCT04230265) employing UniCAR02-T in patients with relapsed AML, ALL, and BPDCN is currently recruiting, and results are awaited. Notably, UniCAR02-T was built-in, combining UniCAR T cells with the specific targeting of a CD123 recombinant antibody derivative molecule (TM123), making it active against its target only in the presence of TM123 [69].

In conclusion, these developments underscore the significant potential of CAR-T therapies, particularly those targeting CD123, in the treatment of hematologic neoplasms, although ongoing clinical trials will provide critical insights into their real-world effectiveness.

Table 1 shows the ongoing and completed clinical trials.

## 5. Anti-CD123 Bispecific Antibodies

Bispecific antibodies have emerged as a groundbreaking therapeutic strategy aimed at concurrently targeting specific antigens on both tumor and immune cells, such as CD3. This innovative class of protein drugs is designed to recruit immune cells to the vicinity of cancer cells, thereby triggering a more targeted and potent immune response. Notably, Blinatumomab, a BsAbs therapy engaging CD19 on leukemia cells and CD3 on T cells, has demonstrated success in treating B-ALL patients [71].

Expanding the scope of BsAbs therapy, researchers are exploring CD123 as a target to redirect the natural immune response toward malignant BPDCN cells (Figure 2). Flotetuzumab (MDG006), a dual-affinity retargeting antibody (DART), has shown promise in redirecting T CD3^+^ lymphocytes against AML cells, displaying potent anti-leukemic activity both in vitro and in vivo [68]. A phase I/II study on relapsed/refractory AML patients revealed noteworthy CR/CR with a partial hematologic recovery (CRh) rate of 26.7% and a median OS of 10.2 months [68]. Ongoing research, including a basket trial evaluating Flotetuzumab in CD123-positive malignancies, is eagerly awaited (NCT04681105).

XmAb14045 represents another potent BsAb targeting both CD123 and CD3, designed for intermittent administration due to its extended serum half-life. In a phase I clinical trial (NCT02730312), XmAb14045 exhibited evidence of anti-leukemic activity, with promising clinical outcomes, including a 23% CR or CR with incomplete hematologic recovery (CR/CRi) at the two highest dose levels [72,73]. However, current data are limited to 104 AML and ALL individuals, necessitating further exploration.

A recent addition to the BsAb landscape is APVO436, a novel anti-CD123 x anti-CD3 BsAb. Preclinical data demonstrate its efficacy both in vitro and in vivo, showcasing superior T cell activation and proliferation, and effective CD123^+^ cell depletion compared to those with MGD006 (Flotetuzumab). Remarkably, APVO436 exhibits enhanced safety with lower T cell cytokine release. In subcutaneous tumor models, it inhibits tumor growth, indicating T cell migration and engagement at the tumor site when human T cells are intravenously implanted. These findings provide a strong rationale for further investigating APVO436 as a potential treatment for AML and other hematological malignancies, including BPDCN [74,75,76]. The unique combination of efficacy and safety profile positions APVO436 as a promising candidate for advancing therapeutic options in these challenging diseases. Future clinical trials will be crucial in validating and expanding upon these encouraging preclinical results, ultimately paving the way for its potential inclusion in the arsenal against hematological malignancies.

Table 1 illustrates ongoing and completed clinical trials.

## 6. Conclusions

BPDCN represents a very rare and aggressive hematologic malignancy, arising from precursors of pDC, behaving like acute myeloid leukemia with high-risk clinical features [3].

Although there has been significant progress in research specifically focused on BPDCN, the development of better treatments remains an urgent priority. In recent times, there has been a notable shift in both clinical and research endeavors toward advancing CD123-targeted immunotherapies for myeloid malignancies, including BPDCN. CD123 stands out as a promising target due to its abnormal expression on BPDCN and AML blasts, distinguishing them from normal hematopoietic stem cells and myeloid progenitors. The success of Tagraxofusp highlights the effectiveness of CD123 targeting in drug development for BPDCN. Such strategies, especially when directed at common antigens like CD123, could broaden the impact of new treatments across various cancers. The continued development of immunotherapies that target CD123 for hematologic malignancies points to additional progress in this field.

The successful clinical translation of SL-401 and CD123CAR T cells has fueled active development in other CD123-targeted immunotherapies, including BsAb, for treating BPDCN and various CD123-related hematologic malignancies. As we await mature results from ongoing studies, it is crucial to consider emergent observations that could impact the efficacy of CD123-targeted immunotherapies to enhance antineoplastic responses and elevate the survival rates for patients dealing with BPDCN and related CD123 neoplasms.

## Figures and Tables

**Table 1:** Clinical trials of CD123-targeting agents.

Study ID	Therapeutic Strategy	Condition/Disease	Phase	Status
**NCT03113643**	***Tagraxofusp*** plus azacitidine ± venetoclax	AML, MDS, BPDCN	I	Recruiting
**NCT04216524**	***Tagraxofusp*** plus azacitidine and chemotherapy	BPDCN	II	Recruiting
**NCT03386513**	***IMGN632***	AML, ALL, BPDCN, MPN	I/II	Active, not recruiting
**NCT04086264**	***IMGN632*** alone or plus azacitidine ± venetoclax	CD123-Positive AML	I/II	Recruiting
**NCT03203369**	Chimeric Antigen Receptor T cells ***UCART 123***	BPDCN	I	Terminated
**NCT04109482**	Chimeric Antigen Receptor T cells ***MB-102***	BPDCN	I/II	Terminated
**NCT02159495**	Chimeric Antigen Receptor T cells ***CD123^+^ CAR T cells***	AML, BPDCN	I	Active, not recruiting
**NCT04230265**	Chimeric Antigen Receptor T cells ***UniCAR02-T + TM123***	AML, BPDCN	I	Recruiting
**NCT04681105**	Bispecific antibodies ***Flotetuzumab***	AML, BPDCN	I	Active, not recruiting

AML: acute myeloid leukemia; MDS: myelodysplastic syndrome; ALL: acute lymphoblastic leukemia; MPN: myeloproliferative neoplasms.

## Data Availability

Not applicable.
